# Stress-Induced Subarachnoid Hemorrhage: A Case Report

**DOI:** 10.7759/cureus.27124

**Published:** 2022-07-21

**Authors:** Charlotte Ritchie, Qamar Al Tinawi, Mostafa Mahmoud Fahmy, Mohammad Selim

**Affiliations:** 1 Department of Medicine, Creighton University School of Medicine, Omaha, USA

**Keywords:** psychological stress, spontaneous subarachnoid hemorrhage, subarachnoid hemmorhage, stress, stress-related cardiomyopathy, sah, internal medicine, takosubo cardiomyopathy, iatrogenic subarachnoid hemorrhage, spontaneous intra-cranial hemorrhage

## Abstract

While there are many forms of intracranial hemorrhage (ICH), the most common form affecting young to middle-aged patients is subarachnoid hemorrhage (SAH). SAHs are primarily traumatic, while a minority of cases are spontaneous. The majority of spontaneous SAHs occur due to the rupture of a cerebrovascular aneurysm. A small number of spontaneous SAHs occur without any objective findings of an aneurysm. Most of these cases are in older patients with certain risk factors such as smoking, hypertension, and alcohol use. This article reports a young female patient without any known significant risk factors who developed an acute spontaneous SAH while experiencing a significant psychological stressor. Recent literature has focused on certain somatic manifestations of psychological stressors, such as stress-induced (Takotsubo) cardiomyopathy. We postulate that our patient’s SAH was a sequela of psychological stress and that the pathophysiology may be similar to Takotsubo cardiomyopathy.

## Introduction

Strokes are the second leading cause of death worldwide and a major cause of disability in adults, impacting not only physical health but also psychological wellness [1]. A recent study reported that 14% of stroke survivors suffer post-stroke depression [2]. Subarachnoid hemorrhages (SAHs) are rare, accounting for only 5% of strokes. Risk factors for SAH include family history, heritable connective tissue disorders, smoking, hypertension, and heavy alcohol use [3,4]. SAHs are divided into two categories: traumatic and spontaneous. Most SAHs are caused by direct trauma to the head (85%). In spontaneous SAHs, 85% are caused by rupture of intracranial aneurysms, and 5% are caused by other structural pathologies (e.g., rupture of an arteriovenous malformation, arterial dissections, tumors, or paragangliomas) [5,6]. No structural abnormality can be identified in 10% of SAHs, which are described as idiopathic or angiogram-negative SAH. In these cases, the hemorrhage is usually confined to the peri-mesencephalic and prepontine cisterns surrounding the midbrain [7]. These SAHs typically occur in the 50th or 60th decade of life and are strongly associated with hypertension [8]. The following describes a case of an angiogram-negative SAH that may have been caused by psychological stress.

## Case presentation

A 34-year-old female presented to the ED complaining of a sudden onset headache with 10/10 severity. The pain started abruptly in the afternoon while she was at work. The headache originated in the front of her head but later wrapped around her head circumferentially and radiated down her neck. She denied any alleviating or aggravating factors. She endorsed nausea, vomiting, and photophobia. However, she denied vision changes, neck rigidity, illnesses or symptoms of fevers, chills, shortness of breath, chest pain, cough, urinary symptoms, hematuria, melena, weakness, or numbness. On physical exam, the patient was supine with her eyes closed. Comprehensive neurology, cardiovascular, pulmonary, and abdominal exams were unremarkable.

The patient’s past medical and surgical history was remarkable for viral meningitis, anemia during pregnancy seven years prior, and tonsillectomy. Her family history was noncontributory. She denied current or past tobacco use and endorsed mild alcohol use (1.0 alcohol units per week). Her only known allergy was kiwi (Actinidia chinensis), and her medications included daily supplemental iron capsules and prenatal vitamins. Of note, she did report extreme stress in her personal life with recent spousal infidelity and subsequent ongoing divorce.

**Figure 1:**
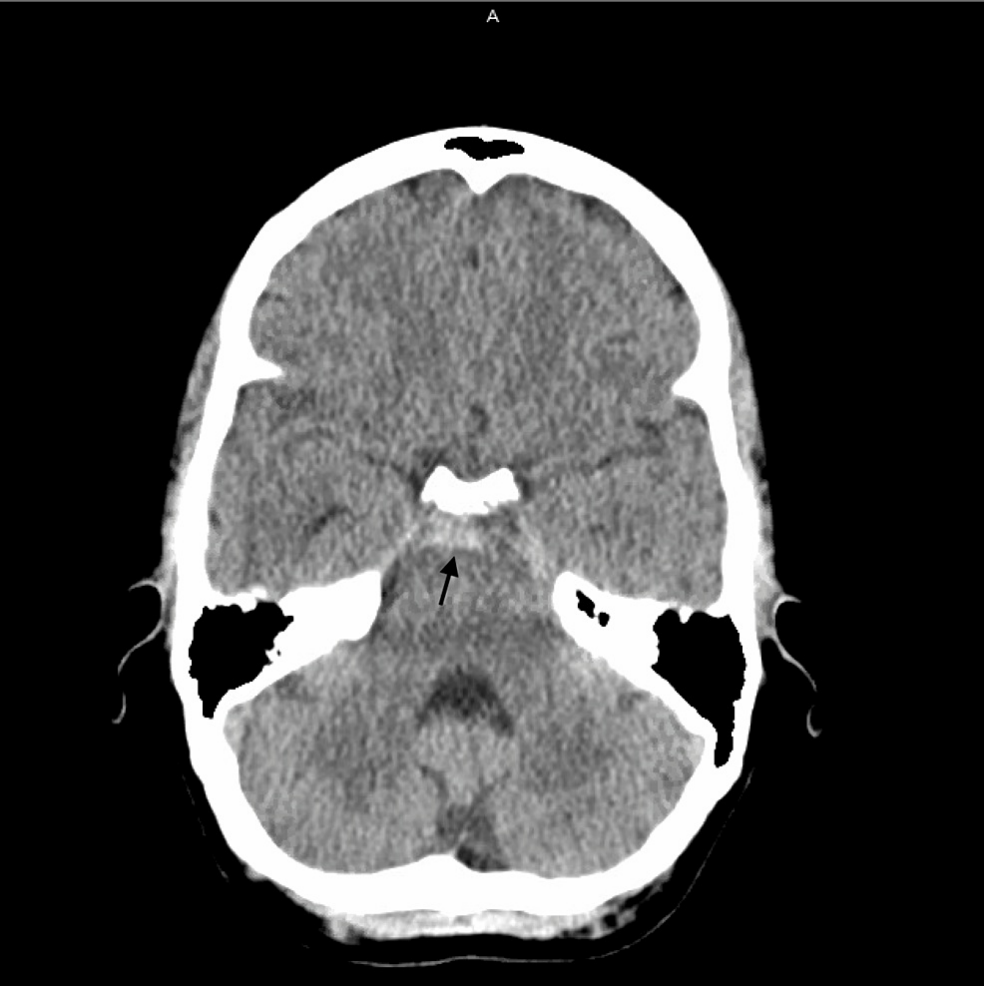
Head CT findings compatible with acute subarachnoid hemorrhage involving portions of the suprasellar, interpeduncular, and prepontine cisterns.

**Figure 2:**
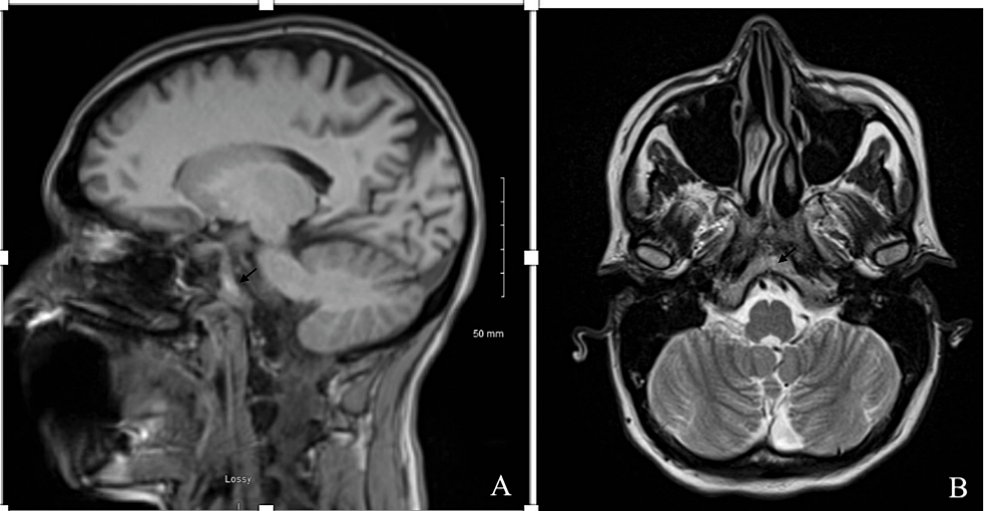
T1-weighted MRI of the brain showing subarachnoid hemorrhage within the basal cisterns in sagittal (A) and axial (B) planes.

**Figure 3:**
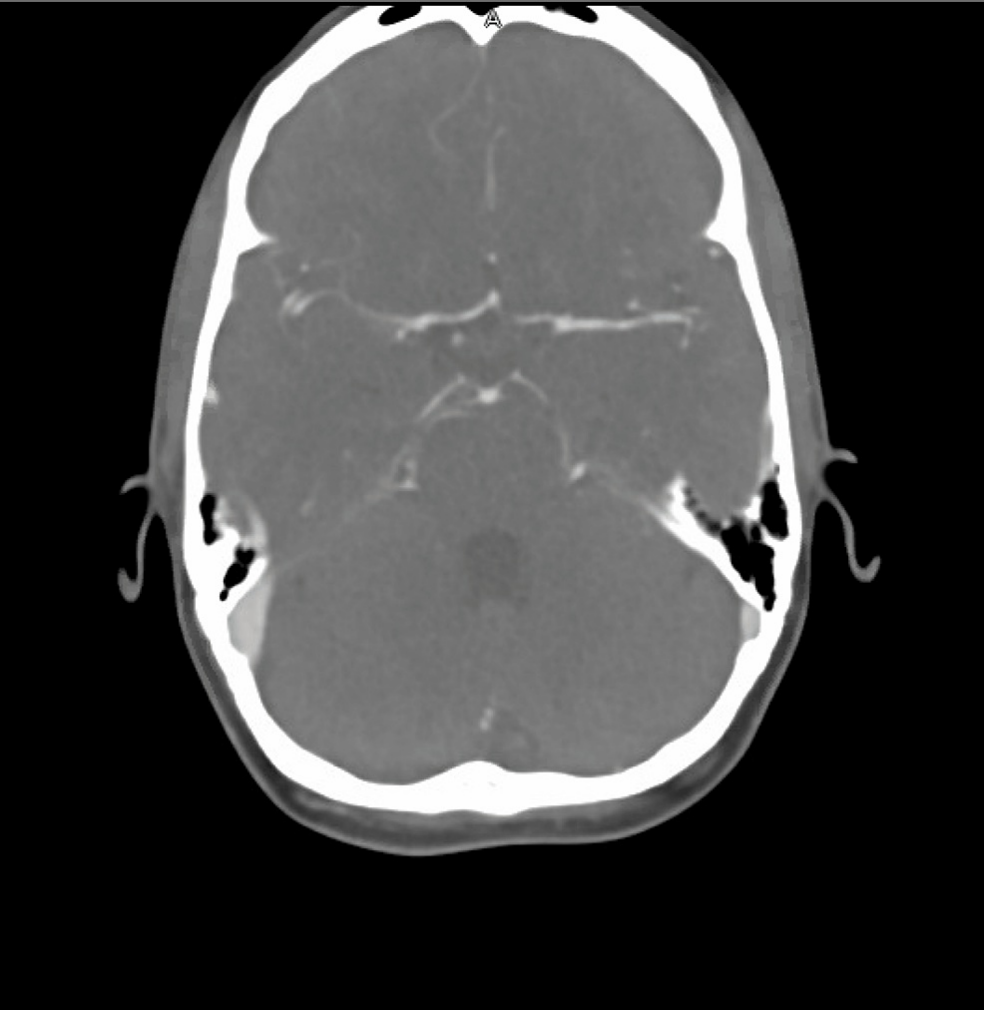
CT angiography of the head and neck showing no evidence of stenosis, large vessel occlusion, or aneurysm.

Labs obtained in the ED were unremarkable. An ECG showed sinus rhythm. A non-contrast CT scan of the head showed an acute SAH involving the suprasellar, interpeduncular, and prepontine cisterns (Figure 1). MRI of the brain confirmed these findings (Figure 2A-2B). CT angiography of the head and neck showed no evidence of stenosis, large vessel occlusion, or aneurysm (Figure 3).

**Figure 4:**
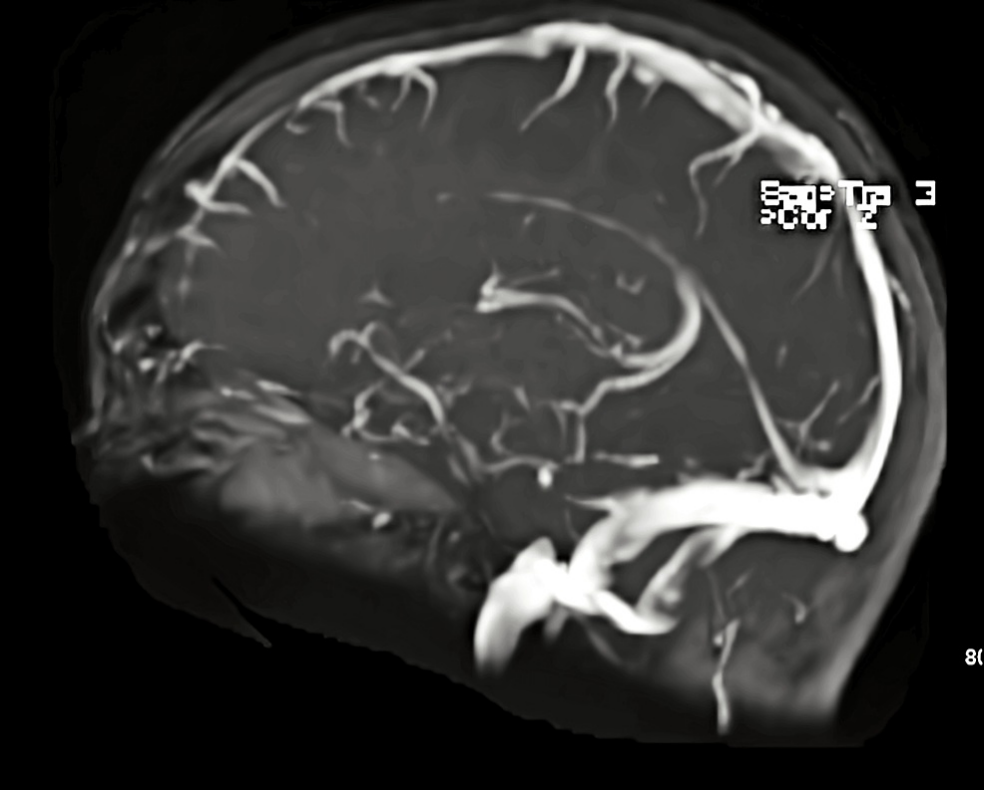
MRI venogram negative for sinus thrombosis.

To discern the cause of her SAH, the patient underwent an MRI venogram without contrast (Figure 4) and an interventional angiogram. Neither image showed any evidence of an aneurysm or structural abnormality. Her blood pressure was tightly controlled in the hospital, and she was started on nimodipine for post-hemorrhagic vasospasm prophylaxis. Nine days after admission, repeat imaging showed no evidence of vasospasm, and her SAH had resolved. For vasospasm, she received a 10-milligram infusion of verapamil into her basilar artery for 11 minutes. She was discharged and prescribed aspirin 81 milligrams once daily and nimodipine 30 milligrams every four hours for 21 days. She followed up two months later with a neurologist and reported no continuing headaches or other neurological, motor, or sensory symptoms.

## Discussion

The case presented above illustrates an example of a potential unique cause of a SAH. The patient’s SAH falls into the category of angiogram-negative SAH, given that the bleed was peri-mesencephalic and no structural abnormalities were found after extensive radiographic imaging. Interestingly, the patient did not share demographical characteristics typical of idiopathic SAHs, nor did she have any current or prior history of high blood pressure. Given the patient’s vitality and absence of structural abnormalities on radiographic imaging, we postulate that the occurrence of acute SAH may have been due to psychological stress. The patient had recently found out that her husband had been having an affair, and she was in the middle of a hostile divorce while trying to secure custody of her two children. Given that the patient was in good physical health with no underlying chronic medical conditions, we suspect this acute increase in mental stress may have caused the SAH. To our knowledge, a case of stress-induced subarachnoid bleeding has not been documented. However, a prior case report described the onset of an acute primary intracerebral hemorrhage that occurred following the patient’s spouse being admitted to the hospital for a severe critical illness. The authors of the case report, as well as another study, suggested that sharp increases in blood pressure due to acute mental stress can cause intracranial vessels to rupture [9,10].

The pathophysiology of stress-induced SAH may be like that of Takotsubo syndrome, a type of cardiomyopathy. The etiology of Takotsubo syndrome is not fully understood but is likely related to an acute release of catecholamines in response to stress. Takotsubo syndrome also shares many similarities with the case presented above. Like our patient, Takotsubo syndrome tends to present in the mid-afternoon and is self-resolving. An arteriogram of the coronary arteries is also unremarkable in Takotsubo syndrome. It has also been reported that Takotsubo syndrome has been triggered by relational conflict and divorce [11]. Additionally, Takotsubo syndrome is a known complication of SAH, indicating that these two pathologies may be closely linked [12].

## Conclusions

This case report suggests psychological stressors as an unusual possible precipitating cause of SAH in a healthy female. Given the absence of underlying risk factors and the temporal relationship between the patient’s acute psychological distress and the SAH, we postulate that psychological stress may have been the inciting cause of this idiopathic or angiogram-negative SAH. Previous cases of ICHs occurring during times of acute psychological distress have been reported, and it is feasible that additional similar events have occurred without being reported. We also propose that the mechanism of a stress-induced SAH may be like that of Takotsubo syndrome, given the similarity in presentation and documented relationship between both pathologies. Additional investigation is needed further to delineate the relationship between psychological stress and ICH.

## References

[1] Donkor ES (2018). Stroke in the 21st century: a snapshot of the burden, epidemiology, and quality of life. Stroke Res Treat.

[2] Kumar R, Kataria N, Kumar N, Kumar M, Bahurupi Y (2020). Poststroke depression among stroke survivors in Sub-Himalayan region. J Family Med Prim Care.

[3] van Gijn J, Kerr RS, Rinkel GJ (2007). Subarachnoid haemorrhage. Lancet.

[4] Feigin VL, Rinkel GJ, Lawes CM, Algra A, Bennett DA, van Gijn J, Anderson CS (2005). Risk factors for subarachnoid hemorrhage: an updated systematic review of epidemiological studies. Stroke.

[5] Macdonald RL, Schweizer TA (2017). Spontaneous subarachnoid haemorrhage. Lancet.

[6] Emami A, Panichpisal K, Benardete E (2011). Clinical reasoning: a rare cause of subarachnoid hemorrhage. Neurology.

[7] Boswell S, Thorell W, Gogela S, Lyden E, Surdell D (2013). Angiogram-negative subarachnoid hemorrhage: outcomes data and review of the literature. J Stroke Cerebrovasc Dis.

[8] Congia S, Carta S, Coraddu M (1994). Subarachnoid hemorrhage of unknown origin. A 44 cases study. Acta Neurol (Napoli).

[9] Lammie GA, Lindley R, Keir S, Wiggam MI (2000). Stress-related primary intracerebral hemorrhage: autopsy clues to underlying mechanism. Stroke.

[10] Caplan L (1988). Intracerebral hemorrhage revisited. Neurology.

[11] Pelliccia F, Kaski JC, Crea F, Camici PG (2017). Pathophysiology of Takotsubo syndrome. Circulation.

[12] Shimada M, Rose JD (2014). Takotsubo cardiomyopathy secondary to intracranial hemorrhage. Int J Emerg Med.

